# *In Vivo* Antimalarial Activity and Toxicity Study of Extracts of *Tagetes erecta* L. and *Synedrella nodiflora* (L.) Gaertn. from the Asteraceae Family

**DOI:** 10.1155/2021/1270902

**Published:** 2021-07-01

**Authors:** Prapaporn Chaniad, Tachpon Techarang, Arisara Phuwajaroanpong, Prasit Na-ek, Parnpen Viriyavejakul, Chuchard Punsawad

**Affiliations:** ^1^Department of Medical Sciences, School of Medicine, Walailak University, Nakhon Si Thammarat 80160, Thailand; ^2^Department of Tropical Pathology, Faculty of Tropical Medicine, Mahidol University, Bangkok 10400, Thailand

## Abstract

**Objective:**

To investigate the antimalarial effects and toxicity of the extracts of the flowers of *Tagetes erecta* L. and the leaves of *Synedrella nodiflora* (L.) Gaertn. in a mouse model.

**Methods:**

To determine the *in vivo* antimalarial activity of the extracts, mice were intraperitoneally injected with the *Plasmodium berghei* ANKA strain and then administered *T. erecta* or *S. nodiflora* extract daily for 4 days. Parasitemia was observed by light microscopy. For the detection of acute toxicity, the mice received a single dose of *T. erecta* or *S. nodiflora* extract and were observed for 14 days. Biochemical parameters of liver and kidney function and the histopathology of liver and kidney tissues of the acute toxicity group were then examined.

**Results:**

*T. erecta* and *S. nodiflora* crude extracts at a dose of 600 mg/kg body weight significantly suppressed parasitemia in malaria-infected mice by 65.65% and 62.65%, respectively. Mice treated with 400 mg/kg *T. erecta* and *S. nodiflora* crude extracts showed 50.82% and 57.67% suppression, and mice treated with 200 mg/kg displayed 26.33% and 38.57% suppression, respectively. Additionally, no symptoms of acute toxicity were observed in the *T. erecta-* and *S. nodiflora*-treated groups. Moreover, no significant alterations in the biochemical parameters of liver and kidney function and no histological changes in the liver or kidney tissues were observed.

**Conclusions:**

This study revealed that both *T. erecta* and *S. nodiflora* extracts have antimalarial properties *in vivo* with less toxic effects. Further studies are needed to elucidate the mechanisms of the active compounds from both plants.

## 1. Introduction

Increasing antimalarial drug resistance is an important crisis that affects the control and elimination of malaria [1]. The development of drug resistance to the current treatments is a global concern. Artemisinin combination therapies **(**ACTs**)** are the first-line drugs for uncomplicated falciparum malaria and consist of a fast-acting artemisinin derivative and a slow-acting partner drug [2]. However, the efficacy of ACTs has encountered problems with delayed parasite clearance, which leads to ACT failure [3–5]. The World Health Organization (WHO) reported an estimated 229 million new infections and 409,000 deaths due to malaria in 2019 [6]. An unsuccessful vaccine and the emergence of resistance to the current drugs have inspired researchers to look for new and effective antimalarial agents with improved efficacy over older drugs. Natural products are a crucial source of many pharmacotherapeutics, and they have acted as template-based compounds for the development of antimalarial drugs [7]. For many years, quinine, a component of the bark of the cinchona tree, was used to treat malaria [8, 9]. The quinine scaffold was then used as a template for modifications to design more potent drug analogs, such as chloroquine and primaquine [8]. Additionally, the powerful antimalarial ingredient artemisinin was extracted and isolated from *Artemisia annua* by Chinese scientists. Artemisinin has also been developed into dihydroartemisinin, artemether, and artesunate as current antimalarial medications [10]. Interestingly, successful antimalarial drugs have been derived from natural plants, and their constituents are suitable for further drug development [11]. Therefore, plant materials are generally sources of inspiration in the search for novel antimalarial agents.

*Tagetes erecta* L**. (***T *. *erecta ***)** and *Synedrella nodiflora ***(**L**.)** Gaertn**. (***S *. *nodiflora*) belong to the Asteraceae family, also called Compositae or sunflower. Asteraceae is one of the largest families of flowering plants, and its members are distributed nearly worldwide but are well represented in temperate and subtropical zones [12]. *T. erecta*, commonly called marigold, quickly germinates, is low maintenance, and contains several benefits, such as natural dyes from the flowers and anti-inflammatory, herbicidal, and antimicrobial activities [13, 14]. The genus *Synedrella* contains only one known species, *S. nodiflora*, which has been used for the treatment of cardiac problems, headaches, earaches, stomachaches, epilepsy, and rheumatism and to stop bleeding [15, 16]. For decades, sesquiterpene lactones were first isolated from plants in the Asteraceae family and are well-known treatments for malaria. Moreover, it has been reported that Asteraceae plants contain various medicinal properties, such as anti-inflammatory, antipyretic, antibacterial, detoxifying, wound healing, antihemorrhagic, antalgic, antispasmodic, and antiparasitic activities [17]. According to our *in vitro* evaluation of the antimalarial activity and toxicity of plants in the Asteraceae family, the aqueous flower extract of *T*. *erecta* and the ethanolic leaf extract of *S*. *nodiflora* have revealed good activity against *P*. *falciparum* without toxicity to Vero cells. Therefore, the present study aimed to confirm the antimalarial activity and toxicity of these extracts in a mouse model.

## 2. Materials and Methods

### 2.1. Collection and Preparation of Plant Samples

The flowers of *T. erecta* and leaves of *S. nodiflora* were collected from Phatthalung Province, Thailand, in March 2020. First, the plant samples were authenticated by Assoc. Prof. Tanomjit Supavita, the School of Pharmacy, Walailak University. Voucher specimens of *S *. *nodiflora ***(**SMD072061001**)** and *T*. *erecta ***(**SMD072063001**)** were deposited at the School of Medicine, Walailak University. The samples were washed with tap water and dried at 60°C in a hot air oven (Memmert, Model; SFE600, Germany**)** for three days and were then powdered with an herb grinder **(**Jincheng, Model; SF, China**).** The different extraction methods were performed with two solvents **(**water and ethanol**).**

#### 2.1.1. Reflux Extraction

The powder of *T. erecta* flowers **(**60 g**)** was extracted with a water solution (600 ml**)** under a reflux system for 2 h. This process was repeated two times. The combined extract solutions were filtered using gauze and Whatman filter paper number 1. The filtrate was then evaporated with a rotary evaporator **(**Rotavapor®, Buchi, China) at 60°C, and the dried extract was stored in a refrigerator at 4°C.

#### 2.1.2. Maceration

The *S *. *nodiflora* leaf powder **(**60 g**)** was soaked in 600 ml of ethanol for 72 h at room temperature. The residue was remacerated twice for another 72 h each time, and the combined extract solutions were filtered through gauze and filter paper. For the drying process, the extract was concentrated with a rotary evaporator as described above. Finally, the dried extract was kept in a refrigerator at 4°C.

### 2.2. Animals and Rodent Parasites

Male ICR mice **(**25 ± 5 g, 4 to 6 weeks of age) were purchased from Nomura Siam International Co., Ltd., Bangkok, Thailand. The animals were housed and acclimated in cages for one week with access to food pellets and clean water ad libitum. The environmental conditions of the laboratory were set to an optimum temperature of 22°C **(**±3°C**)** and 55–60**%** humidity. The animals were maintained on a 12 h light/dark cycle. Good hygiene was maintained by the animal care staff to clean and remove feces from the cages daily. This study obtained approval from the Walailak University Ethical Review Committee and was in accordance with the National Guidelines for Handling Laboratory Animals **(**clearance no. WU-AICUC-63-014**).** The wild-type *Plasmodium berghei* ANKA strain was obtained through BEI Resources, NIAID, NIH**: ***P *. *berghei*, strain ANKA, MRA-311, contributed by Thomas F. McCutchan.

### 2.3. Four-Day Suppressive Test

The details of the four-day suppressive test were described by Peters [18], and this test was used to determine schizonticidal activity. First, the mice were randomly divided into eight groups of five mice each. Group I served as a negative control and received 7**%** Tween 80 and 3**%** ethanol in distilled water, which was the vehicle used for the dissolved crude extracts and drug. Group II served as a positive control, where the mice received 6 mg/kg artesunate. Groups III, IV, and V served as treatment groups and received the aqueous extract of *T. erecta* at doses of 200, 400, and 600 mg/kg, respectively. Finally, groups VI, VII, and VIII received the ethanolic extract of *S. nodiflora* at doses of 200, 400, and 600 mg/kg, respectively. The extracts were administered to each group orally by gavage. In brief, the mice were injected intraperitoneally with 1 × 10^7^*P*. *berghei*-infected red blood cells. Administration of the extract to be tested began 3 h after infection (D0) and was continued daily for 3 consecutive days (24, 48, and 72 h after infection**).** Blood from the tail vein was collected to prepare thin films on day 4. The thin blood films were stained with 10**%** Giemsa solution, the infected red blood cells (iRBCs) were examined under a light microscope **(**Olympus, Model; CX-31, Japan**)** by counting three different fields with approximately 300 red blood cells per field, and the average of the results was then utilized to calculate percent parasitemia using the following formula**:**

(1) $$\begin{array}{c} \%\text{ parasitemia}=\frac{\text{number of infected red blood cells}}{\text{number of total red blood cells }}\times 100. \end{array}$$

The percent parasitemia suppression was determined using the following formula**:**

(2) $$\begin{array}{c} \%\text{ suppression}=\frac{\left[A-B\right]}{A}\times 100, \end{array}$$

where *A* is the average percentage of parasitemia in the negative control group and *B* is the average percentage of parasitemia in the extract-treated group.

### 2.4. Acute Toxicity

Twenty ICR mice were randomly divided into four groups of five mice per group, including mice treated with 2000 mg/kg *T. erecta* extract, mice treated with 2000 mg/kg *S *. *nodiflora* extract, mice treated with 7**%** Tween 80 **(**as a negative control**)**, and an untreated control group. Acute oral toxicity was evaluated in the mice according to the guideline procedures outlined by the Organization for Economic Co-operation and Development (OECD) in 2008 [19]. The animals were fasted for 3 h before the beginning of the experiment. Both crude extracts **(***T*. *erecta* and *S*. *nodiflora ***)** were dissolved in a 7**%** Tween 80 solution to make stock solutions at a concentration of 2000 mg/kg. A single high dose of 2000 mg/kg body weight of each extracted plant was then administered to each experimental group, whereas 7**%** Tween 80 solution was administered to the negative control group. The mice were observed in detail for any indications of toxic effects, including rigidity, sleep, diarrhea, depression, abnormal secretion, and hair erection, within the first 30 min after administration and then daily for a period of 14 days. At the end of the experiment, all mice were anesthetized by intraperitoneal injection with Nembutal solution **(**pentobarbital sodium**) (**Ceva Sante Animale, Maassluis, Netherlands**).** Blood samples for biochemical analyses were then collected by using a Vacuette lithium heparin tube. The liver and kidney tissues were harvested and fixed in a 10**%** formalin solution for histopathological examination.

### 2.5. Biochemical Analyses

Whole blood samples were collected by the cardiac puncture technique. After centrifugation of the whole blood at 3,000 g for 5 min, the plasma was used to investigate liver and kidney function. Aspartate aminotransferase (AST**)**, alanine aminotransferase (ALT**)**, and alkaline phosphatase (ALP**)** were tested as the biochemical parameters of liver function. Kidney function was evaluated based on the plasma levels of blood urea nitrogen (BUN**)** and creatinine. All biochemical parameters were determined by using an AU480 chemistry analyzer **(**Beckman Coulter, USA**).**

### 2.6. Histopathological Examination

Histopathological observations were performed according to standard laboratory procedures [20, 21]. Briefly, the mice (five mice/group) at the end of the experiment (day 14) were sacrificed, and specimens were collected. Both liver and kidney tissues were fixed in 10% (v/v) formalin at room temperature for 24–48 h. After fixation, the tissues were embedded in paraffin and serially sliced into 5 *µ*m thick sections using a manual rotary microtome (Model RM2235, Leica Biosystems, Germany). Each section was deparaffinized in xylene, rehydrated with a series of ethanol solutions, stained with hematoxylin and eosin (H&E) solution, and mounted with glass coverslips. The histopathological changes were investigated under a light microscope by two independent observers who were blinded to the experimental groups.

### 2.7. Statistical Analysis

Statistical analysis was carried out using SPSS statistical software version 21 **(**SPSS, IL, USA**).** Each experimental value was compared with its corresponding control. All quantitative data are expressed as the means ± standard errors of the mean (SEMs). The normality of distribution was tested by using the Kolmogorov–Smirnov test. Statistical significance of the mean parasitemia suppression and biochemical parameters between groups was carried out by one-way analysis of variance (ANOVA**)** followed by post hoc Tukey**'**s multiple comparison test. Statistical significance was set at *p* < 0.05 for all tests.

## 3. Results

### 3.1. Antimalarial Activity

The aqueous extract of *T. erecta* and ethanolic extract of *S. nodiflora* were examined for their antimalarial properties by using a 4-day suppressive test. The animals in each group received a daily oral dose of the plant extracts at different concentrations (200, 400, and 600 mg/kg body weight). The results showed that the *T. erecta* and *S. nodiflora* extracts effectively suppressed *Plasmodium* parasites compared with the negative control group, especially at high doses. The parasite counts decreased in a dose-dependent manner after treatment with the aqueous extract of *T. erecta* and the ethanolic extract of *S. nodiflora*. However, there was not complete suppression after treatment with any of the *T. erecta* and *S. nodiflora* extract-treated groups, whereas the 6 mg/kg body weight artesunate-treated group, the positive control group, suppressed the parasites by more than 93%. The percentages of parasitemia and suppression are summarized in Table 1.

### 3.2. Acute Toxicity

Mice were treated with a single dose of 2000 mg/kg *T. erecta* aqueous extract or of *S. nodiflora* ethanolic extract on the first day of the experiment. Then, physical and behavioral changes were observed daily after treatment for 14 days. We found that there were no notable symptoms, such as hair erection, feeding changes, vomiting, diarrhea, abnormal secretion, abnormal sleep, or excitement that would indicate toxicity during the experimental period. No mortality was observed in any of the mice within the first 24 h or for the following 14 days. Therefore, the lethal doses of the *T. erecta* and *S. nodiflora* extracts seem to be greater than 2000 mg/kg body weight.

### 3.3. Effects of the *T. erecta* and *S. nodiflora* Extracts on Liver and Kidney Function

In the 2000 mg/kg aqueous *T. erecta* extract treatment group, the biochemical parameters of the liver, including AST, ALT, and ALP, were observed in plasma. There was no significant difference in these parameters compared with the untreated control and 7% Tween 80 groups (*p* < 0.05). In addition, the levels of BUN and creatinine, as the biochemical parameters of kidney function, in the mice treated with 2000 mg/kg *T. erecta* extract showed no significant difference from those in the untreated control and 7% Tween 80 groups. Similar to aqueous *T. erecta* extract treatment, the biochemical parameters of both liver and kidney function in mice treated with 2000 mg/kg *S. nodiflora* ethanolic extract showed no significant difference when compared with the untreated control and 7% Tween 80 groups (*p* < 0.05) as shown in Table 2. These results indicate that neither the aqueous extract of *T. erecta* nor the ethanolic extract of *S. nodiflora* disturbed the functions of the liver or kidney.

If the data are not significantly different, a marker indicating significance is not shown.

### 3.4. Histopathological Examination of the Livers and Kidneys of the Mice

**Figure 1:**
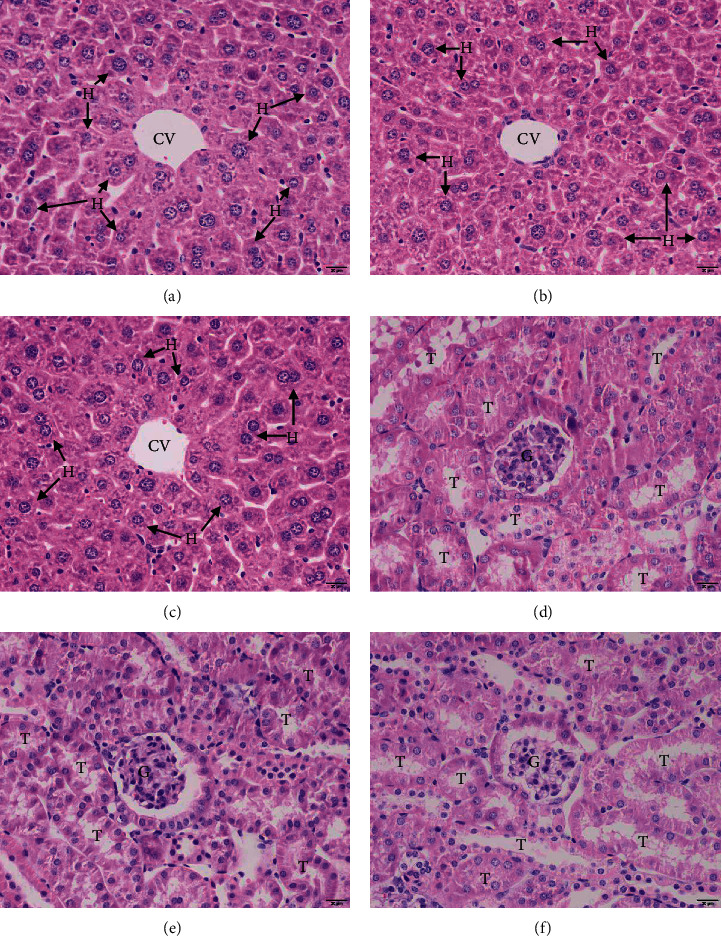
Histopathological changes in the livers and kidneys of the untreated control group (a, d), 7% Tween 80 group (b, e), and 2000 mg/kg aqueous *T. erecta* extract-treated group (c, f). All images are of 400x magnification. Bars = 20 *μ*m. T, tubules; G, glomerulus; CV, central vein; H, hepatocyte.

The histopathological images of the livers and kidneys of the mice treated with the aqueous extract of *T. erecta* are illustrated in Figure 1. The results showed normal histology in the livers of mice treated with 2000 mg/kg *T. erecta* extract, for example, in the morphological characteristics of the hepatocytes, such as size, arrangement, and color staining. Moreover, sinusoidal vasodilatation and inflammatory cell infiltration were not detected (Figure 1(c)) when compared with the untreated control (Figure 1(a)) and 7% Tween 80 (Figure 1(b)) groups. Histopathological changes in the kidney were also investigated. The histological features of the kidneys of this group were also normal, including normal glomeruli, Bowman's capsules, and kidney epithelial cells (Figure 1(f)) compared with those in the untreated control (Figure 1(d)) and 7% Tween 80 (Figure 1(e)) groups.

**Figure 2:**
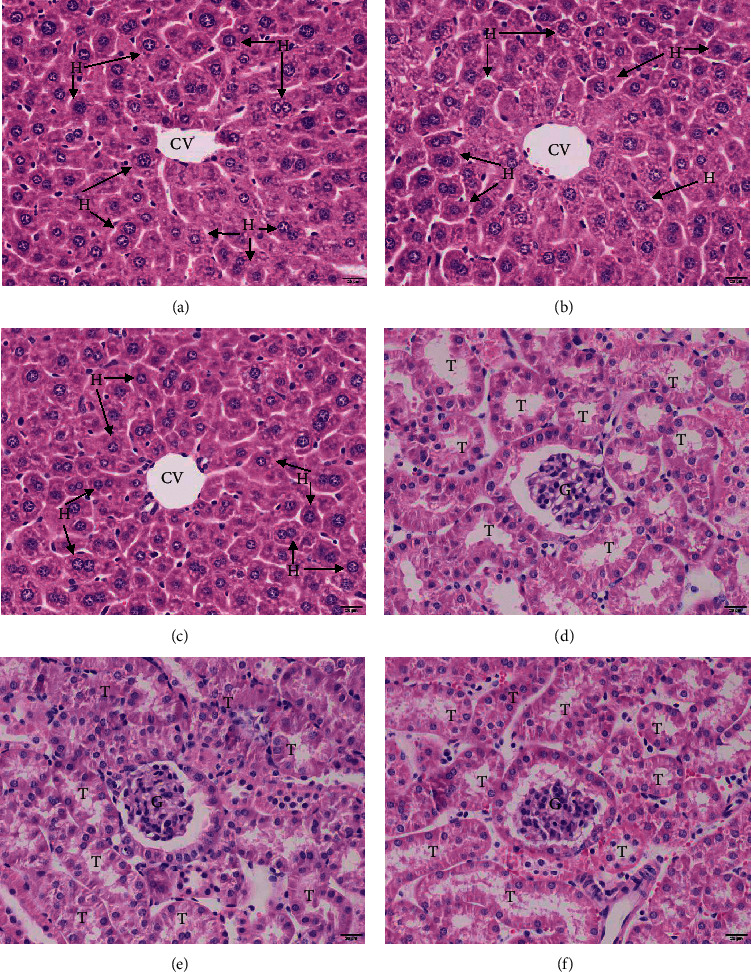
Pathological changes in the livers and kidneys of the untreated control group (a, d), 7% Tween 80 group (b, e), and 2000 mg/kg ethanolic *S. nodiflora* extract-treated group (c, f). All images are of 400x magnification. Bars = 20 *μ*m. T, tubules; G, glomerulus; CV, central vein; H, hepatocyte.

The histopathological changes in the livers and kidneys of mice treated with the ethanolic extract of *S. nodiflora* are shown in Figure 2. No histopathological changes were observed in the livers of the 2000 mg/kg *S. nodiflora*-treated group (Figure 2(c)), such as the morphology, size, arrangement, and color staining of hepatocytes, sinusoid capillaries, and inflammatory cell infiltration in the liver when compared to those of the untreated control (Figure 2(a)) and 7% Tween 80 groups (Figure 2(b)). The kidneys of the mice in the 2000 mg/kg *S. nodiflora*-treated group were also observed. There was also normal histopathology in the kidneys of this treatment group, such as the morphology of the glomeruli, Bowman's capsules, and kidney epithelial cells (Figure 2(f)) compared with the untreated control (Figure 2(d)) and 7% Tween 80 groups (Figure 2(e)).

## 4. Discussion

During the process of drug discovery from natural sources, *in vitro* and *in vivo* studies are performed to evaluate of efficacy and safety of a new drug candidate [22]. Our previous *in vitro* screen of the antimalarial activity of plants in the Asteraceae family found that aqueous extracts of *T. erecta* and *S. nodiflora* exerted antimalarial effects against the *P. falciparum* K1 strain, which is a chloroquine-resistant strain, with IC_50_ values of 35.61 and 37.82 *µ*g/ml, respectively.

Accordingly, these two plants were considered for further evaluation of their *in vivo* antimalarial properties in this study. An *in vivo* model was established to evaluate their possible prodrug effects, the potential involvement of the immune system in the eradication of infection, and the safety of the drug before progression into clinical trials [23, 24]. In the present study, ICR mice were infected with wild-type *P. berghei* ANKA as a model for the investigation of antimalarial activity. *P. berghei* ANKA infection is commonly used for the induction of malaria in a mouse model that can be subjected to a 4-day suppressive test. This strain is the parasite of choice due to its ability to sequester within the microcirculation, which is a characteristic of severe malaria [25]. Importantly, this parasite strain has been used for the identification of several antimalarial agents, including chloroquine, mefloquine, halofantrine, and artemisinin derivatives [26]. We used the 4-day suppressive test because it is the commonly used method for evaluating the antimalarial activity of candidate agents in early infection. In this method, the percent suppression of blood parasitemia is the most reliable parameter [23].

Other previous studies on *T. erecta* extracts found that the ethanolic *T. erecta* extract shows highly effective antifeedant, insecticidal, and growth inhibitory activity against *Papilio demoleus* Linnaeus [27]. The chemical components of *T. erecta* have various properties, including antimicrobial, insecticidal, herbicidal, antioxidant, carbon tetrachloride-induced hepatic injury protection, wound healing, and antipain effects in acetic acid-induced mice [28], and effects on decreasing blood sugar levels [29]. The antimalarial properties of *T. erect*a extract in an animal model were found to show high effectiveness in a dose-dependent manner. A high dose of the aqueous *T. erecta* extract (600 mg/kg body weight) exhibited a 65.65% decrease in *P. berghei*. The phytochemical screening of the aqueous flower extract of *T. erecta* showed the presence of flavonoids, tannins, and saponins, which is in agreement with the compounds found in other reports [30]. Therefore, the antimalarial activity of the aqueous *T. erecta* extract might be due to the effects of flavonoids. A previous study reported that this class of compounds exhibits antimalarial effects by inhibiting *Plasmodium* fatty acid synthesis and the influx of L-glutamine and myoinositol into infected erythrocytes [31]. The major compound in *T. erecta* was found to be quercetagetin (89.91%), which is a flavonol (a type of flavonoid) [32]. Thus, this compound might be responsible for the antimalarial effects of the *T. erecta* extract. Other components found in *T. erecta* included syringic acid, methyl-3,5-dihydroxy-4-methoxy benzoate [33], limonene, terpinolene, (Z)-myroxide, piperitone, piperitenone, piperitenone oxide, and *β*-caryophyllene [29].

The determination of acute toxicity *in vivo* is recommended as the first step in drug discovery to exclude compounds that are toxic to the vertebrate host [34]. Therefore, the acute toxicity of the aqueous extract of *T. erecta* was investigated in ICR mice. The animals received a single dose of 2000 mg/kg *T. erecta* extract and were observed for symptoms of toxicity for 14 days. All of the treated mice survived until the end of the experiment. In addition, biochemical analyses were performed to examine the functions of the liver and kidney. The levels of AST and ALT depend on the destruction of cells. Both AST and ALT can be used to indicate abnormalities in the liver and heart [35, 36]. We found that the levels of AST and ALT were not different between the treated and control groups. In addition to kidney function, the levels of BUN and creatinine were measured, as both BUN and creatinine can be excreted by the kidneys. In the case of kidney damage or renal dysfunction, the highest levels of BUN and creatinine would be found in the blood. Increases in these levels may be caused by reduced blood supply to the kidneys or an obstruction in the urinary tract, resulting in the inability to excrete BUN and creatinine normally [37]. We found that the aqueous *T. erecta*-treated group showed normal levels of BUN and creatinine that were similar to those in the control group. Moreover, the histological features of the liver and kidneys of mice treated with 2000 mg/kg *T. erecta* extract were normal. Therefore, the aqueous extract of *T. erecta* at 2000 mg/kg body weight did not cause acute toxicity in this animal model. Subacute toxicity has been reported in mice that received a *T. erecta* chloroform extract at doses of 200 and 400 mg/kg body weight. However, the results of this study showed no toxicity symptoms in the mice. Moreover, all biochemical parameters showed normal levels when compared with the control group. These results indicated that there was no difference between the treated and control groups [38].

The inhibitory action of the ethanolic *S. nodiflora* extract on malaria-infected mice showed that the extract produced dose-dependent suppression. The maximum parasite suppression of 62.65% was observed at the highest dose (600 mg/kg). Additionally, the percent parasite suppression was 57.67% and 38.57% at the 400 and 200 mg/kg doses, respectively. Based on the classification in a previous study [39], the *S. nodiflora* extract is considered to have moderate inhibitory activity against *P. berghei* infection. According to the phytochemical constituent analysis, alkaloids are present in the leaf extract of *S. nodiflora*, and these compounds have been reported as potential antimalarial agents [40]. Therefore, we believe that the compounds that are active against malaria parasites might be alkaloids.

A safety assessment was used to determine the harmfulness of this plant. After the mice were treated with the ethanolic extract of *S. nodiflora*, there were no observable changes in their normal behavior and no symptoms of toxicity. In toxicology, the LD_50_ value is the dose of a test substance required to kill half of the tested population, and this value is typically obtained from acute toxicity studies [41]. The crude ethanolic extract of *S. nodiflora* has an LD_50_ greater than 2000 mg/kg body weight because no mouse deaths were observed during the experiment. In addition, the effect of the extract on liver-kidney functions was determined by histopathological analysis. An increase in AST, ALT, and ALP values indicates liver damage [35, 36], and a rise in BUN and creatinine levels suggests a failure of the kidneys or their possible malfunction [37]. This study found no significant difference in the functions of either the livers or kidneys in the treatment group when compared to the control group. Additionally, histopathological analysis of the liver and kidney did not reveal any abnormalities. Therefore, this result suggests that oral administration of the ethanolic extract of *S. nodiflora* is safe. Information obtained herein on the toxicological effects of this extract agrees with previous studies. The hydroethanolic extract of *S. nodiflora* has a low toxicity profile after long-term continuous oral administration and is safe in subacute toxicity tests [42, 43].

As previously mentioned, the two medicinal plants used in this study, *T. erecta* and *S. nodiflora*, belong to the Asteraceae family, and the plants in this family constitute excellent natural sources of antimalarial agents. *Artemisia annua* is a traditional Chinese plant containing artemisinin, a potent antimalarial drug [44]. Other plants belonging to the same family, such as *Xanthium strumarium* L., *Bidens pilosa* L., and *Eclipta alba* (L.) Hassk, also reportedly exhibit antimalarial activity [17].

## 5. Conclusions

This study provides the first report of the antimalarial activity of the aqueous extract of *T. erecta* flowers and the ethanolic extract of *S. nodiflora* leaves in a mouse model. The results from this investigation revealed that both extracts exhibit promising antimalarial activity against *P. berghei* infection with no adverse health effects on the liver or kidney. Further studies are needed to elucidate the mechanisms of the active compounds from both plants.

## Figures and Tables

**Table 1:** Effects of aqueous extract of *T. erecta* and ethanolic extract of *S. nodiflora* against *Plasmodium* parasites in malaria-infected mice.

Group	Dose (mg/kg)	iRBCs	% parasitemia	% suppression
Negative control	—	7.50 ± 1.91	64.25 ± 0.57^b,c,d,e,f,g,h^	—

Artesunate	6	192.75 ± 1.71	3.64 ± 0.59^a,c,d,e,f,g,h^	95.82 ± 2.71^c,d,e,f,g,h^

*T. erecta* extract	200	142.00 ± 2.92	47.33 ± 0.97^a,b,d,e^	26.33 ± 1.51^b,d,e^
	400	94.80 ± 3.27	31.60 ± 0.98^a,b,c,e^	50.82 ± 1.52^b,c,e^
	600	66.20 ± 1.48	22.07 ± 0.49^a,b,c,d^	65.65 ± 0.77^b,c,d^

*S. nodiflora* extract	200	118.40 ± 3.51	39.47 ± 1.17^a,b,g,h^	38.57 ± 1.82^b,g,h^
	400	81.60 ± 6.11	27.20 ± 2.04^a,b,f,h^	57.67 ± 3.17^b,f,h^
	600	72.00 ± 3.61	24.00 ± 1.20^a,b,f,g^	62.65 ± 1.87^b,f,g^

The data are presented as the means ± SEMs (*n* = 5 per group), *p* < 0.05. iRBCs: the number of infected red blood cells per field; the total number of RBCs per field was 300. ^a^Compared with the negative control, ^b^compared with artesunate, ^c^compared with the 200 mg/kg *T. erecta* extract, ^d^compared with the 400 mg/kg *T. erecta* extract, ^e^compared with the 600 mg/kg *T. erecta* extract, ^f^compared with the 200 mg/kg *S. nodiflora* extract, ^g^compared with the 400 mg/kg *S. nodiflora* extract, and ^h^compared with the 600 mg/kg *S. nodiflora* extract.

**Table 2:** Effects of the *T. erecta* aqueous extract and *S. nodiflora* ethanolic extract on the biochemical parameters of liver and kidney function in the acute toxicity test.

Group	Liver function test		
	AST (U/L)	ALT (U/L)	ALP (U/L)
Untreated control	124.06 ± 8.53	37.20 ± 2.77	97.31 ± 6.99
7% Tween 80	128.30 ± 4.85	33.75 ± 3.56	98.52 ± 4.51
*T. erecta* extract	125.56 ± 10.75	32.30 ± 5.53	101.58 ± 6.21
*S. nodiflora* extract	120.03 ± 7.26	33.78 ± 3.42	91.38 ± 2.36

Group	Kidney function test		
	BUN (mg/dL)	BUN (mg/dL)	

Untreated control	23.94 ± 0.44	0.54 ± 0.03	
7% Tween 80	22.27 ± 1.30	0.56 ± 0.03	
*T. erecta* extract	23.78 ± 0.93	0.54 ± 0.01	
*S. nodiflora* extract	22.22 ± 0.72	0.56 ± 0.01	

The data are presented as the means ± SEMs (*n* = 5 per group), *p* < 0.05.

## Data Availability

The data used to support the findings of this study are included within the article.

## References

[1] Menard D., Dondorp A. (2017). Antimalarial drug resistance: a threat to malaria elimination. *Cold Spring Harbor Perspectives in Medicine*.

[2] Li Q., Pybus B. (2019). *Pharmacokinetic and Pharmacodynamic Profiles of Rapid- and Slow-Acting Antimalarial Drugs, Malaria*.

[3] Thanh N., Nguyen T.-N., Tuyen N. (2017). Rapid decline in the susceptibility of *Plasmodium falciparum* to dihydroartemisinin–piperaquine in the south of Vietnam. *Malaria Journal*.

[4] Fairhurst R. M., Dondorp A. M. (2016). Artemisinin-resistant *Plasmodium falciparum* malaria. *Microbiology Spectrum*.

[5] Nsanzabana C. (2019). Resistance to artemisinin combination therapies (ACTs): do not forget the partner drug!. *Tropical Medicine and Infectious Disease*.

[6] WHO (2019). *World Malaria Report*.

[7] Fernández-Álvaro E., Hong W. D., Nixon G. L., O’Neill P. M., Calderón F. (2016). Antimalarial chemotherapy: natural product inspired development of preclinical and clinical candidates with diverse mechanisms of action. *Journal of Medicinal Chemistry*.

[8] Jones R. A., Panda S. S., Hall C. D. (2015). Quinine conjugates and quinine analogues as potential antimalarial agents. *European Journal of Medicinal Chemistry*.

[9] Achan J., Talisuna A. O., Erhart A. (2011). Quinine, an old anti-malarial drug in a modern world: role in the treatment of malaria. *Malaria Journal*.

[10] Sharma C., Kumar S. (2015). *Recent Advances in Antimalarial Drug Discovery-Challenges and Oppportunities*.

[11] Lahlou M. (2013). The success of natural products in drug discovery. *Pharmacology & Pharmacy*.

[12] Islam M., Rahman A. H. M. (2016). An assessment of the family Asteraceae at Shadullapur Upazila of Gaibandha District, Bangladesh with particular reference to medicinal plants. *Journal of Progressive Research in Biology (JPRB)*.

[13] Laosinwattana C., Wichittrakarn P., Teerarak M. (2018). Chemical composition and herbicidal action of essential oil from *Tagetes erecta* L. leaves. *Industrial Crops and Products*.

[14] Singh Y., Gupta A., Kannojia P. (2020). *Tagetes erecta* (marigold) - a review on its phytochemical and medicinal properties. *Current Medical and Drug Research*.

[15] Amoateng P., Woode E., Kombian S. B. (2012). Anticonvulsant and related neuropharmacological effects of the whole plant extract of *Synedrella nodiflora* (L.) Gaertn (Asteraceae). *Journal of Pharmacy & Bioallied Sciences*.

[16] Idu M., Onyibe H. I. (2007). Medicinal plants of Edo state, Nigeria. *Research Journal of Medicinal Plant*.

[17] Panda S. K., Luyten W. (2018). Antiparasitic activity in Asteraceae with special attention to ethnobotanical use by the tribes of Odisha, India. *Parasite*.

[18] Peters W., Portus J. H., Robinson B. L. (1975). The chemotherapy of rodent malaria, XXII. *Annals of Tropical Medicine & Parasitology*.

[19] OECD (2008). *Test No. 425: Acute Oral Toxicity: Up-and-Down Procedure*.

[20] Chaniad P., Techarang T., Phuwajaroanpong A., Punsawad C. (2019). Antimalarial activity and toxicological assessment of *Betula alnoides* extract against *Plasmodium berghei* infections in mice. *Evidence-Based Complementary and Alternative Medicine*.

[21] Phuwajaroanpong A., Chaniad P., Horata N., Muangchanburee S., Kaewdana K., Punsawad C. (2020). *In vitro* and *in vivo* antimalarial activities and toxicological assessment of *Pogostemon cablin* (Blanco) Benth. *Journal of Evidence-Based Integrative Medicine*.

[22] Brake K., Gumireddy A., Tiwari A., Chauhan H., Kumari D. (2017). *In vivo* studies for drug development via oral delivery: challenges, animal models and techniques. *Pharmaceutica Analytica Acta*.

[23] Mekonnen L. B. (2015). *In vivo* antimalarial activity of the crude root and fruit extracts of *Croton macrostachyus* (Euphorbiaceae) against *Plasmodium berghei* in mice. *Journal of Traditional and Complementary Medicine*.

[24] Haddadi M., Mousavi M. J., Mohseni S., Mardani G. (2020). *In vitro* ADME screening instead of *in vivo* studies in preclinical safety. *Biomedical Journal of Scientific & Technical Research (BJSTR)*.

[25] Basir R., Rahiman S. F., Hasballah K. (2012). *Plasmodium berghei* ANKA infection in ICR mice as a model of cerebral malaria. *Iranian Journal of Parasitology*.

[26] Mekuria A. B., Geta M., Birru E. M., Gelayee D. A. (2021). Antimalarial activity of seed extracts of *Schinus molle* against *Plasmodium berghei* in Mice. *Journal of Evidence-Based Integrative Medicine*.

[27] Matintarangsan N., Wattanuruk D. (2017). Effect of marigold extract (*Tagetes erecta* L.) in controlling the *Papilio demoleus* Linnaeus (Lepidoptera: papilionidae). *VRURDI*.

[28] Priyanka D., Shalini T., Navneet V. K. (2013). A brief study on marigold (*Tagetes* species): a review. *International Research Journal of Pharmacy*.

[29] Krishna A., Kumar S., Mallavarapu G. R., Ramesh S. (2004). Composition of the essential oils of the leaves and flowers of *Tagetes erecta* L.. *Journal of Essential Oil Research*.

[30] Dasgupta N., Ranjan S., Shree M., Saleh M. A. A. M., Ramalingam C. (2015). Blood coagulating effect of marigold (*Tagetes erecta* L.) leaf and its bioactive compounds. *Oriental Pharmacy and Experimental Medicine*.

[31] Ntie-Kang F., Onguéné P., Lifongo L. L., Ndom J., Sippl W., Mbaze L. (2014). The potential of anti-malarial compounds derived from African medicinal plants, part II: a pharmacological evaluation of non-alkaloids and non-terpenoids. *Malaria Journal*.

[32] Wang W., Xu H., Chen H., Tai K., Liu F., Gao Y. (2016). In vitro antioxidant, anti-diabetic and antilipemic potentials of quercetagetin extracted from marigold (*Tagetes erecta* L.) inflorescence residues. *Journal of Food Science and Technology*.

[33] Phrutivorapongkul A., Kiattisin K., Jantrawut P., Chansakaow S., Vejabhikul S., Leelapornpisid P. (2013). Appraisal of biological activities and identification of phenolic compound of African marigold (*Tagetes erecta*) flower extract. *Pakistan Journal of Pharmaceutical Sciences*.

[34] Romanha A. J., Castro S. L. D., Soeiro M. D. N. C. (2010). *In vitro* and *in vivo* experimental models for drug screening and development for Chagas disease. *Memórias Do Instituto Oswaldo Cruz*.

[35] Anosike C., Ugwu U., Nwakanma O. (2010). Effect of ethanol extract of *Pyrenacantha Staudtii* leaves on carbontetrachloride induced hepatotoxicity in rats. *Biokemistri*.

[36] Nigatu T. A., Afework M., Urga K., Ergete W., Makonnen E. (2017). Toxicological investigation of acute and chronic treatment with *Gnidia stenophylla* Gilg root extract on some blood parameters and histopathology of spleen, liver and kidney in mice. *BMC Research Notes*.

[37] Hassanalilou T., Payahoo L., Shahabi P. (2017). The protective effects of *Morus nigra* L. leaves on the kidney function tests and histological structures in streptozotocin-induced diabetic rats. *Biomed Res (Aligarh)*.

[38] Nikkon F., Habib M., Saud Z., Karim M., Roy A., Zaman S. (2009). Toxicological evaluation of chloroform fraction of flower of *Tagetes erecta* L. on rats. *International Journal of Drug Development & Research*.

[39] Zeleke G., Kebebe D., Mulisa E., Gashe F. (2017). *In vivo* antimalarial activity of the solvent fractions of fruit rind and root of *Carica papaya* Linn (Caricaceae) against *Plasmodium berghei* in mice. *Journal of Parasitology*.

[40] Uzor P. F. (2020). Alkaloids from plants with antimalarial activity: a review of recent studies. *Evidence-Based Complementary and Alternative Medicine*.

[41] Gad S. C. (2014). *LD50/LC50 (Lethal Dosage 50/Lethal Concentration 50)*.

[42] Amoateng P., Adjei S., Osei-Safo D. (2016). Long-term continuous administration of a hydro-ethanolic extract of *Synedrella nodiflora* (L) Gaertn in male Sprague-Dawley rats: biochemical, haematological and histopathological changes. *Ghana Medical Journal*.

[43] Adjei S., Amoateng P., Osei-Safo D. (2014). Sub-acute toxicity of a hydro-ethanolic whole plant extract of *Synedrella nodiflora* (L) Gaertn in rats. *International Journal of Green Pharmacy*.

[44] Weathers P. J., Arsenault P. R., Covello P. S., McMickle A., Teoh K. H., Reed D. W. (2011). Artemisinin production in *Artemisia annua*: studies in planta and results of a novel delivery method for treating malaria and other neglected diseases. *Phytochemistry Reviews*.

